# Supplementary material for: Nuclear Functions of KaeA, a Subunit of the KEOPS Complex in Aspergillus nidulans
Source: Int J Mol Sci. 2022 Sep 22;23(19):11138. doi: 10.3390/ijms231911138 (PMC9570407; doi:10.3390/ijms231911138)
Supplement: Supplementary file 1 [file ijms-23-11138-s001.zip › ijms-1835910-supplementary/Figure S1.pdf]

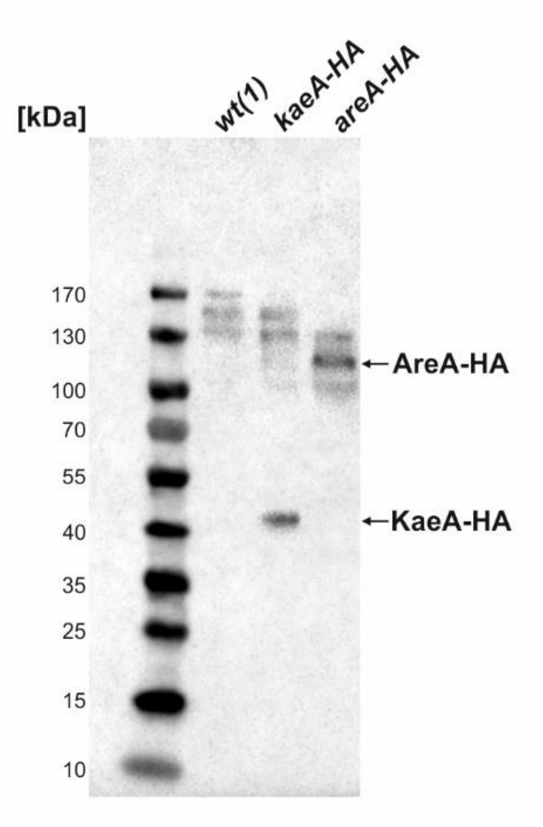

Figure S1. Western analysis of protein extracts from strains expressing KaeA-HA (7) and AreA-HA (9) fusions. The positions of fusion proteins are indicated.
