# Supplementary material for: Nuclear Functions of KaeA, a Subunit of the KEOPS Complex in Aspergillus nidulans
Source: Int J Mol Sci. 2022 Sep 22;23(19):11138. doi: 10.3390/ijms231911138 (PMC9570407; doi:10.3390/ijms231911138)
Supplement: Supplementary file 1 [file ijms-23-11138-s001.zip › ijms-1835910-supplementary/Table S1.pdf]

Table S1 – Primer sequences

A. Primers for RT-qPCR

AN gene number	Forward primer	Reverse primer
**AN0948**	CTGATAGGTCGCAATGGCAC	CGCATAACCGTCTTCACTAG
**AN10854**	TCTCCGTCTGTGGTCACGAG	GCGATCGGAGGTAAGAGGAG
**AN1298**	CTCCGGCATTGATACAGATG	AGATCTGCGACATATGGCTC
**AN1812**	CGAACGCACCACAGCTCTTC	CGAACGCACCACAGCTCTTC
**AN1848**	TAGTGTTGCCGCCATCCACC	TCATAGCAAGCGTGGCATCC
**AN2366**	CCTGAATACAACGCCAACAC	CTCAGAAGGAAGCGAAGAAG
**AN5170**	GCAGCAGAACATCGTAGATC	CTTGTTGGCGTTGAGAATGC
**AN6076**	CCAAGAACGCCGACATGCTC	CCATGCGTGGACGGTAGATC
**AN8168**	CGCAGGTCCATTCACTCAAG	TATTGATGAAGGCGAGGTGC

B. Primers for primer-extention

**Name**	**sequence**
IletRNArev	GGGGGTCGAACCCTCGAC
IletRNArev-yeast	GGGATCGAACCGCTGATC
ValtRNArev	CGGGCTCGATCCGGCGACC

C. Primers for cloning

**Golden Gate for obtaining KaeA::GFP fusion**
**KaeA**
**F: TTTACCTGCTAATGGCGCCATCGGTCTTGAAGGTTC**

**R: TTTACCTGCGCCGATCTCTCCATTGCACAAATAC**
**GFPPyr**
**F: TTTACCTGCTATAAGATGGAGCTGGTGCAGGCGCTG**
**R: TTTACCTGCGATACTGTCTGAGAGGAGGCACTGATG**
**DF1**
**F: TTTACCTGCGCGCACAGACTTATTCAGCCATATCCTG**
**R: TTTACCTGCAGTCATTGCGCTTCATATCCGCCTATC**
**DF2:**
**F: TTTACCTGCCGTGCAATACAGGTGTCTTGGACAAGGCTTC**
**R: TTTACCTGCTTATGGCGCCGATGAAGGATGATAGCAGTC**
**Overlap extension PCR for obtaining KaeA::TEV::GFP fusion**
**First step**
**F: TTGCCCAGGCTGGTATGCTCGCATATAAGACAGGCTTCCGGACGCCGCTC**
**R: GGCACCGGCTCCAGCGCCTGCACCAGCTCCGTGGTGCCCAGCGGATTGGA**
**Second step**
**5'-TTGCCCAGGCTGGTATGCTCGCATATAAGACAGGCTTCCGGACGCCGCTCAAGGAATCTACGTGCACGCAGCGCTTCCGGACGGATGATGTATTTGTGCAATGGAGAGATGAGAACCTGTACTTCCAATCCGCTGGGCACCACGGAGCTGGTGCAGGCGCTGGAGCCGGTGCC-3'**
**Overlap extension PCR for obtaining KaeA::HA**
**First step**
**F: CGGACGGATGATGTATTTGTGCAATGGAGAGATTACCCATACGATGTTCC**
**R: AGAGGGTGAAGAGCATTGTTTGAGGCGACCGGTAGCGTAATCTGGAACGT**
**Second step**
**5'-CGGACGGATGATGTATTTGTGCAATGGAGAGATTACCCATACGATGTTCCTGACTATGCGGGCTATCCGTATGACGTCCCGGACTATGCAGGATCCTATCCATATGACGTTCCAGATTACGCTACCGGTCGCCTCAAACAATGCTCTTCACCCTCT-3'**

D. Primers for ChiP-Seq qPCR control experiment

**Name**	**sequence**
niiA–niaDfw	CGGGAACTGGGAACTGTCC
niiA–niaDrev	GATTCCGAGGCTCTGATTGG
