# Supplementary material for: Nuclear Functions of KaeA, a Subunit of the KEOPS Complex in Aspergillus nidulans
Source: Int J Mol Sci. 2022 Sep 22;23(19):11138. doi: 10.3390/ijms231911138 (PMC9570407; doi:10.3390/ijms231911138)

Table S11 – Potential phosphorylation sites in KaeA

Results of the *A. nidualns* KaeA scan with NetPhos-3.1. The NetPhos 3.1 server predicts serine, threonine or tyrosine phosphorylation sites in eukaryotic proteins using ensembles of neural networks. Both generic [77] and kinase specific predictions [78] were performed. Stringent setting were used: for each residue only the best prediction and only the scores higher than 0.5 are shown.

Position of the phosphorylated amino acid residue	Phosphorylated aminno acid Residue	Sequence context of the phosphorylation site	Score	Kinase
25	S	PKDGSTPQV	0.628	PKA
26	T	KDGSTPQVL	0.511	p38MAPK
36	T	NIRHTYVSP	0.770	PKC
37	Y	IRHTYVSPP	0.749	unsp
39	S	HTYVSPPGE	0.928	unsp
50	T	LPKDTARHH	0.589	unsp
60	S	SWVVSLVKK	0.508	PKC
72	S	EARISVDDV	0.997	unsp
81	Y	DCICYTKGP	0.538	INSR
102	S	ARTLSLLWG	0.676	PKA
126	T	GRLITGASN	0.719	PKC
129	S	ITGASNPVV	0.512	cdc2
135	Y	PVVLYVSGG	0.682	unsp
141	T	SGGNTQVIA	0.511	unsp
148	S	IAYSSQRYR	0.791	PKC
151	Y	SSQRYRIFG	0.596	unsp
172	T	RFARTLHIS	0.538	PKC
183	Y	PAPGYNIEQ	0.527	EGFR
200	Y	VDLPYTVKG	0.611	unsp
237	T	EEDVTDVTP	0.519	CKII
240	T	VTDVTPVSD	0.799	unsp
243	S	VTPVSDGAL	0.921	unsp
249	S	GALESRKPT	0.977	unsp
253	T	SRKPTRADL	0.801	PKA
260	S	DLCFSLQET	0.547	unsp
311	S	DRGGSVHAT	0.996	unsp
337	T	LAYKTGFRT	0.728	PKC
341	T	TGFRTPLKE	0.551	cdk5
346	S	PLKESTCTQ	0.916	unsp
349	T	ESTCTQRFR	0.847	PKC
354	T	QRFRTDDVF	0.573	unsp

Graphical representation of phosphorylation sites in KaeA:

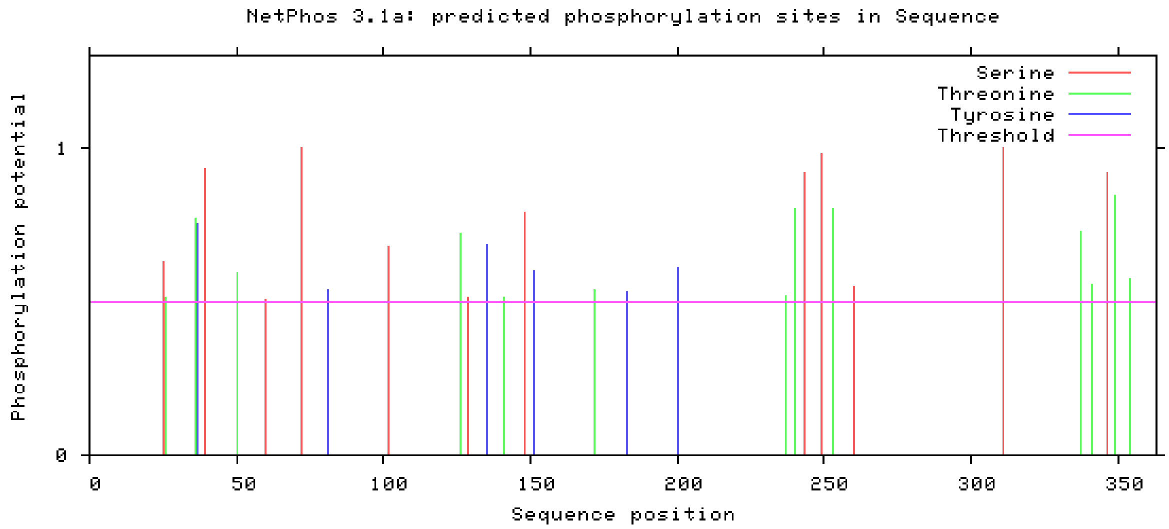

Supplement: Supplementary file 1 [file ijms-23-11138-s001.zip › ijms-1835910-supplementary/Table S11.pdf]
