# Supplementary material for: Nuclear Functions of KaeA, a Subunit of the KEOPS Complex in Aspergillus nidulans
Source: Int J Mol Sci. 2022 Sep 22;23(19):11138. doi: 10.3390/ijms231911138 (PMC9570407; doi:10.3390/ijms231911138)
Supplement: Supplementary file 1 [file ijms-23-11138-s001.zip › ijms-1835910-supplementary/Table S7.pdf]

Table S7 - Alignment summary data for ChIP-Seq analysis

Sample	Quality control passed reads	Secondary reads	Mapped reads	Properly paired reads
JG_Input1_FR	20 603 923	7 427	18 598 914 (90.27%)	18 396 952 (89.32%)
JG_Input2_FR	23 824 824	9 706	21 409 760 (89.86%)	21 078 070 (88.51%)
JG_Input3_FR	13 852 838	5 500	12 552 649 (90.61%)	12 406 246 (89.59%)
JG_IP1_FR	18 683 924	3 924	17 783 487 (95.18%)	17 688 710 (94.69%)
JG_IP2_FR	12 864 888	2 672	12 390 476 (96.31%)	12 321 010 (95.79%)
JG_IP2_FR	14 186 414	3 572	13 372 157 (94.26%)	13 315 084 (93.88%)
